# Genetic variations in olfactory receptor gene *OR2AG2* in a large multigenerational family with asthma

**DOI:** 10.1038/s41598-019-54718-6

**Published:** 2019-12-13

**Authors:** Samarpana Chakraborty, Pushkar Dakle, Anirban Sinha, Sangeetha Vishweswaraiah, Aditya Nagori, Shivalingaswamy Salimath, Y. S. Prakash, R. Lodha, S. K. Kabra, Balaram Ghosh, Mohammed Faruq, P. A. Mahesh, Anurag Agrawal

**Affiliations:** 1 Centre of Excellence for Translational Research in Asthma and Lung Diseases, CSIR-Institute of Genomics and Integrative Biology, New Delhi, India; 2 Academy of Scientific and Innovative Research (AcSIR), Chennai, India; 3 Genetics and Genomics Lab, Department of Genetics and Genomics, University of Mysore, Manasagangotri, Mysuru, Karnataka India; 4 Department of Pulmonary Medicine, JSS Medical College, JSSAHER, Mysore, Karnataka India; 5 Departments of Anesthesiology, Physiology and Biomedical Engineering, Mayo Clinic, Rochester, Minnesota USA; 6 Department of Pediatrics, All India Institute of Medical Sciences, New Delhi, India

**Keywords:** Next-generation sequencing, Respiration

## Abstract

It is estimated from twin studies that heritable factors account for at-least half of asthma-risk, of which genetic variants identified through population studies explain only a small fraction. Multi-generation large families with high asthma prevalence can serve as a model to identify highly penetrant genetic variants in closely related individuals that are missed by population studies. To achieve this, a four-generation Indian family with asthma was identified and recruited for examination and genetic testing. Twenty subjects representing all generations were selected for whole genome genotyping, of which eight were subjected to exome sequencing. Non-synonymous and deleterious variants, segregating with the affected individuals, were identified by exome sequencing. A prioritized deleterious missense common variant in the olfactory receptor gene *OR2AG2* that segregated with a risk haplotype in asthma, was validated in an asthma cohort of different ethnicity. Phenotypic tests were conducted to verify expected deficits in terms of reduced ability to sense odors. Pathway-level relevance to asthma biology was tested in model systems and unrelated human lung samples. Our study suggests that *OR2AG2* and other olfactory receptors may contribute to asthma pathophysiology. Genetic studies on large families of interest can lead to efficient discovery.

## Introduction

Asthma is a chronic disorder of the airways caused by a strong genetic predisposition, in addition to environmental insults^1^. Previous genetic studies in asthma such as linkage analysis, twin studies, candidate gene approaches and GWAS (Genome Wide Association Studies), have identified numerous candidate genes and variants for asthma, but all the associated variants can explain less than 15% of the genetic risk^2^. Thus, a large piece of the puzzle is missing. The gap may be reduced by gaining information of genetic variants that are missed by the population studies, but may have strong effects in closely related individuals that share the variants. Discovery of such highly penetrant variants associated with asthma-risk is more efficient through the study of closely related individuals, such as multi-generation large families, rather than population based studies. Next generation sequencing has shown a promise in identifying novel targets which were previously missed by the candidate gene based approaches^3^. Exome sequencing i.e. sequencing the coding regions of the genome in a familial set up has proven to be highly informative in the context of other complex diseases such as cardiovascular diseases and Alzheimer’s disease^4,5^

Earlier, in Indian population, using candidate gene and genome wide candidate gene approaches, several genes including those for cytokines, FcεR1, STAT6 and INPP4A were found to be associated with asthma^6,7^. In India, there exists a rich reservoir of large families owing to endogamy and the concept of compound families. Also, cases of consanguinity and inter-clan marriages provide an excellent ground for well-designed genetic studies. Since asthma is a complex and heterogeneous disorder, we hypothesized that a study design that can enable identification of highly penetrating genetic variants could be achieved by studying large families with multiple generations affected with asthma. For this, a subset of subjects belonging to a four generation family from southern India with clinical history of asthma and atopy over four generations were selected for exome sequencing. In addition to this, all available samples from the family were subjected to whole genome genotyping for linkage and haplotype analysis.

Post bioinformatics analysis of exome sequencing and sequencing validation, a genetic variant from one candidate gene *OR2AG2*, encoding an olfactory receptor, was found in our multi-generational family to be novel in asthma. Literature suggests the expression of olfactory receptors on the airways similar to bitter taste receptors, and having roles in mucociliary clearance on activating stimuli by strong odor^8^. Studies in recent past, including a GWAS in a Korean population reported significant importance of olfactory receptors with respect to lung function^9^. We hypothesized that defect in sensing and clearance by these receptors may trigger exacerbations, which is a commonly observed phenomenon in asthmatics in presence of strong/volatile odor. Olfactory receptors having a pertinent role in the human lungs is a relatively new concept. We, therefore, explored the putative role of olfactory receptor gene, *OR2AG2* in the context of allergic asthma using *in vitro* systems and human lung samples.

## Results

### Exome sequencing and analysis pipeline

**Figure 1:**
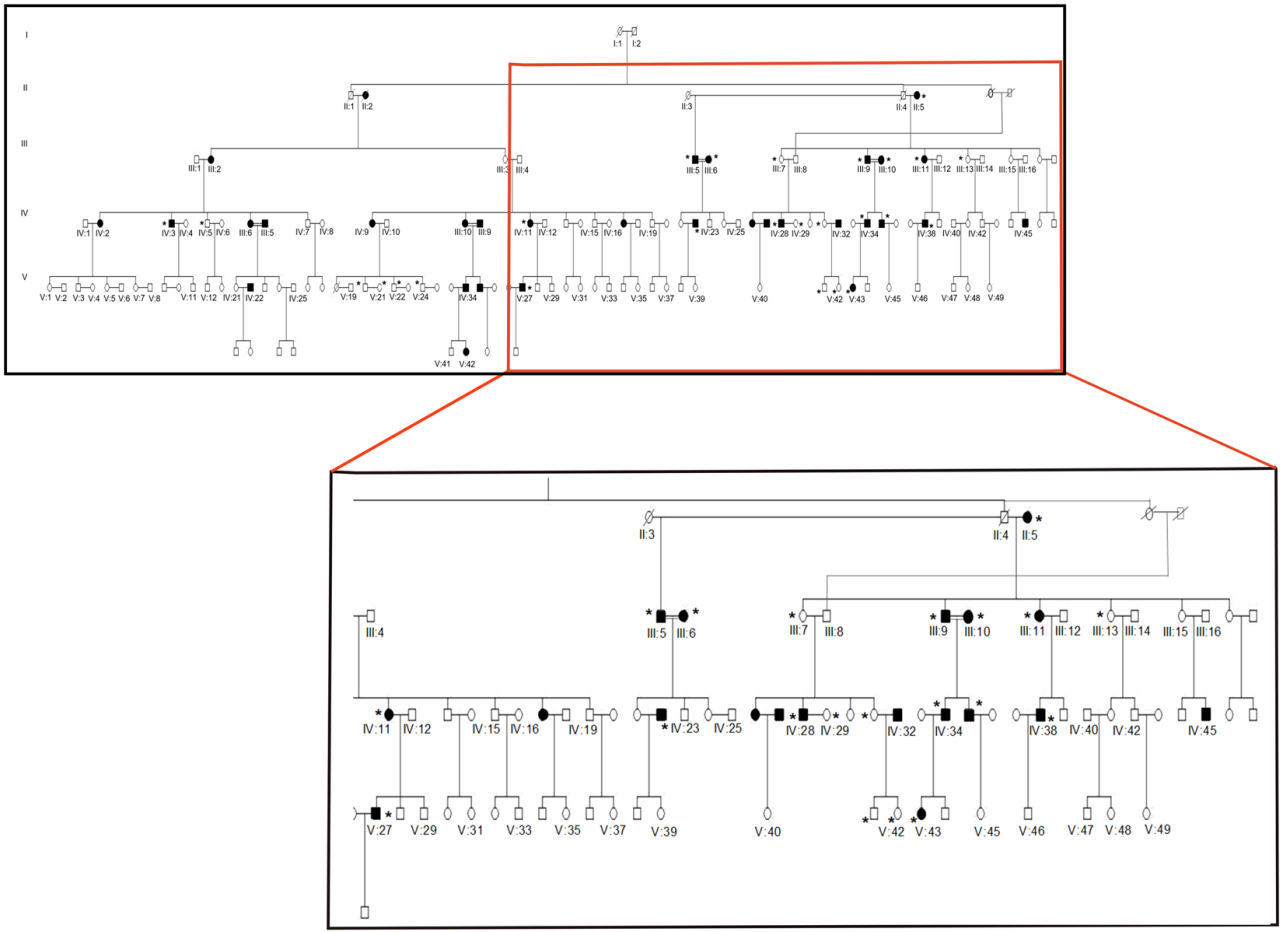
Pedigree and sample information of family 1. Family 1 is a four generation family with approximately 40% individuals affected with asthma and atopy. Samples collected are marked with * symbol. Black square/circle denotes affected members while clear square/circle denotes unaffected subjects. No clear inheritance pattern could be identified from the pedigree. Interestingly, this family also has cases of consanguinity (depicted by double-lines=). Eight subjects- II:5, III:7, III:9, III:10, IV:31, IV:34, V:27, V:42 (five cases and three controls) were selected for Exome Sequencing.

A four generation pedigree was constructed with information provided by the family (Fig. 1). Clinical details of participating subjects are provided in Table 1. Eight samples were subjected to exome sequencing using the Illumina platform. A couple with consanguinity along with a distant member of the family were chosen for sequencing since their shared genome will be less, thus providing higher chances of finding variants that truly segregate with the disease.

**Table 1:** Clinical details of subjects participating in the study from family 1.

	Asthma		Control	
Age (Mean Year ± SEM)	50 ± 5.2		45.16 ± 6.6	
Gender (M/F)	(9/5)		(3/3)	
Weight (kg) (Mean ± SEM)	67 ± 4.2		62.3 ± 7.4	
No. of Smokers	2		2	
	Pre-BD	Post-BD	Pre-BD	Post-BD
Mean FEV1(L)	1.63	1.77	1.76	1.82
Mean FVC (L)	2.23	2.34	2.01	2.19
Mean FEV1/FVC	0.7	0.72	0.82	0.82
Mean zFEV1	−3.74	−3.55	−3.41	−3.12
% BDR_FEV1	7.90		3.40	
% BDR_FVC	4.93		8.95	

Pre/post BD: Pre/post bronchodilation, FEV1 : Forced expiratory volume in one second, FVC : Forced vital capacity, % BDR : Percent bronchodilator responsiveness. The spirometry data has been analysed using lung function equations for Indian population^34^.

Data obtained after sequencing was subjected to an analysis pipeline (Supplementary Fig. E1A). The average coverage of sequencing was ≈50X. A total of 115463 variants were identified, post annotation. We aimed at identifying variants that segregated with only affected subjects and were absent in unaffected subjects. For this, a model free approach was used, since asthma is a complex disease and the pedigree showed a multifarious mode of disease inheritance. 910 variants were seen to segregate with the affected members of the family. These variants were further subjected to prioritization, before technical validation.

### Variant prioritization strategies

**Figure 2:**
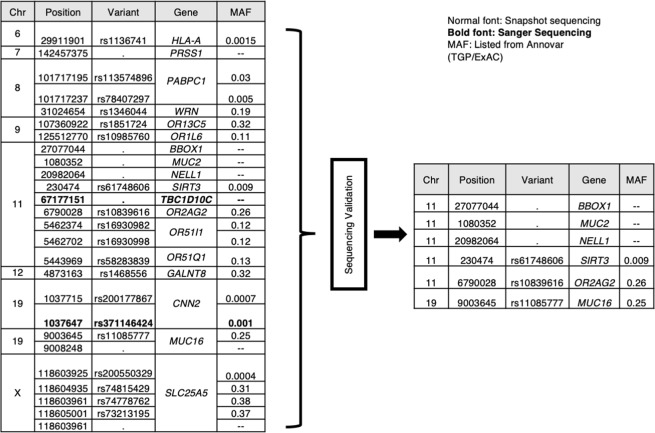
List of variants for validation of Family 1. The figure shows the list of novel variants (left) that were subjected to validation in additional members from family 1 to filter out false positives from exome sequencing, using Sanger (in italics) and SNaPshot sequencing reactions, (MAF: Minor allele frequency). The list of validated variants is shown in right.

To understand the possible implication of all the identified segregating variants, the following parameters were used: 1. In-silico prediction tools (explained in Supplementary and Supplementary Fig. E1B) were exploited to understand the possible implication of the variants in human physiology and disease, and the ones predicted to have damaging consequences were considered. 2. Genetic variants from genes unreported in asthma (novel) that co-segregated with the disease were given priority. The idea to focus on these variants was in concordance with our initial hypothesis that a fraction of missing heritability for asthma is attributable to deleterious and highly penetrating genetic variants that are more likely to be identified in a large family based set-up. In-silico predicted deleterious variants that segregated only with asthma cases and belonged to novel genes of asthma were prioritized and validated using Sanger sequencing. Sanger sequencing based confirmation was conducted on all available samples from Family 1. Indels were confirmed by Sanger sequencing of the region surrounding the variant of interest, while SNPs were validated by SNaPshot sequencing. Fig. 2 shows the list of variants from 18 genes selected for Sanger sequencing and the final list of confirmed variants that co-segregated with the disease. GWAS database identified 5 of these variants in previously published asthma and lung reports (Details are provided in Supplementary Text and Table E3).

Of the confirmed variants, one common variant in the gene *OR2AG2*, rs10839616, (NM_001004490.1:c.161 G > C) was found to belong to a risk haplotype that co-segregated with all the affected members of the large family, as observed by whole genome genotyping of all the subjects of the family (Supplementary Fig. E3). In addition to this, whole genome genotyping study was also performed on an ongoing cohort of 141 pediatric-asthma cases with 130 controls for which we had clinical data **(**Tables 2 and 3**)**. This information was then used to check if any of the disease co-segregating variants were associated with asthma in the general population. In line with the idea that missing variants may have small effects at a population level, lost during statistical corrections in small underpowered studies, we used unadjusted associations for the intersection. Out of the six disease co-segregating variants, the *OR2AG2* genetic variant rs10839616 was found to be significantly associated with asthma (OR = 1.579, p = 0.0132; MAF = 0.42). The variant rs10839616 was also observed in a previously reported GWAS on pulmonary function parameters (Supplementary Table E3).

**Table 2:** Age (in years) and gender details of human subjects participating in case control cohort of asthma.

Subjects	Males	Females	Age (Mean years)
Control	83	47	32.6
Asthmatic	115	26	8.6

**Table 3:** Clinical characteristics of genotyped samples from the pediatric cohort of asthma. (FEV1 : Forced Expiratory Volume in one second,  FVC : Forced vital capacity, MEF25: Maximum expiratory flow at 25% of FVC,  MEF75 :  Maximum expiratory flow at 75% of FVC, PEFR : Peak expiratory flow rate, A: ANOVA, K: Kruskal Wallis).

Variable	GG (n = 57)	GC (n = 58)	CC (n = 26)	Statistical Test
	Mean (SD)	Mean (SD)	Mean (SD)	p-value *(A/K)
Age (Months)	106.88 (42.09)	101.36 (38.20)	106.50 (38.36)	0.73 (A)
Exhaled Nitric Oxide (PPB)	24.65 (19.66)	18.89 (11.95)	16.77 (9.56)	0.28 (K)
FEV1 (% predicted)	89.2 (18.76)	83.28 (19.02)	83.33 (17.70)	0.27 (A)
FVC (% predicted)	86.96 (15.99)	81.93 (16.34)	82.10 (17.48)	0.30 (A)
MEF25 (% predicted)	79.43 (48.19)	77.86 (40.62)	89.57 (48.24)	0.75 (K)
MEF75 (% predicted)	91.58 (29.69)	84.32 (30.25)	84.67 (29.83)	0.47 (A)
PEFR (% predicted)	74.48 (20.65)	67.43 (24.14)	69.05 (20.07)	0.29 (A)
Exacerbation Frequency	0.13 (0.14)	0.20 (0.22)	0.16 (0.12)	0.30 (K)
Smell Test PEA	8.4 (3.09)	7.04 (3.39)	6.48 (3.63)	0.19 (A)

Supplementary Fig. E4A shows the impact of *OR2AG2* genetic variation on the protein sequence of *OR2AG2*. Multiple sequence alignment depicts conservation of the wild type amino acid across different species of non-human primates suggesting the evolutionary importance of the variant of interest (Fig. E4B). Furthermore, in silico prediction suggests possible loss of function of the *OR2AG2* gene in the affected subjects carrying the genetic variant, rs10839616 (c.161 G > C) (Fig. E4C).

### Phenotypic correlation of genotypic findings

**Figure 3:**
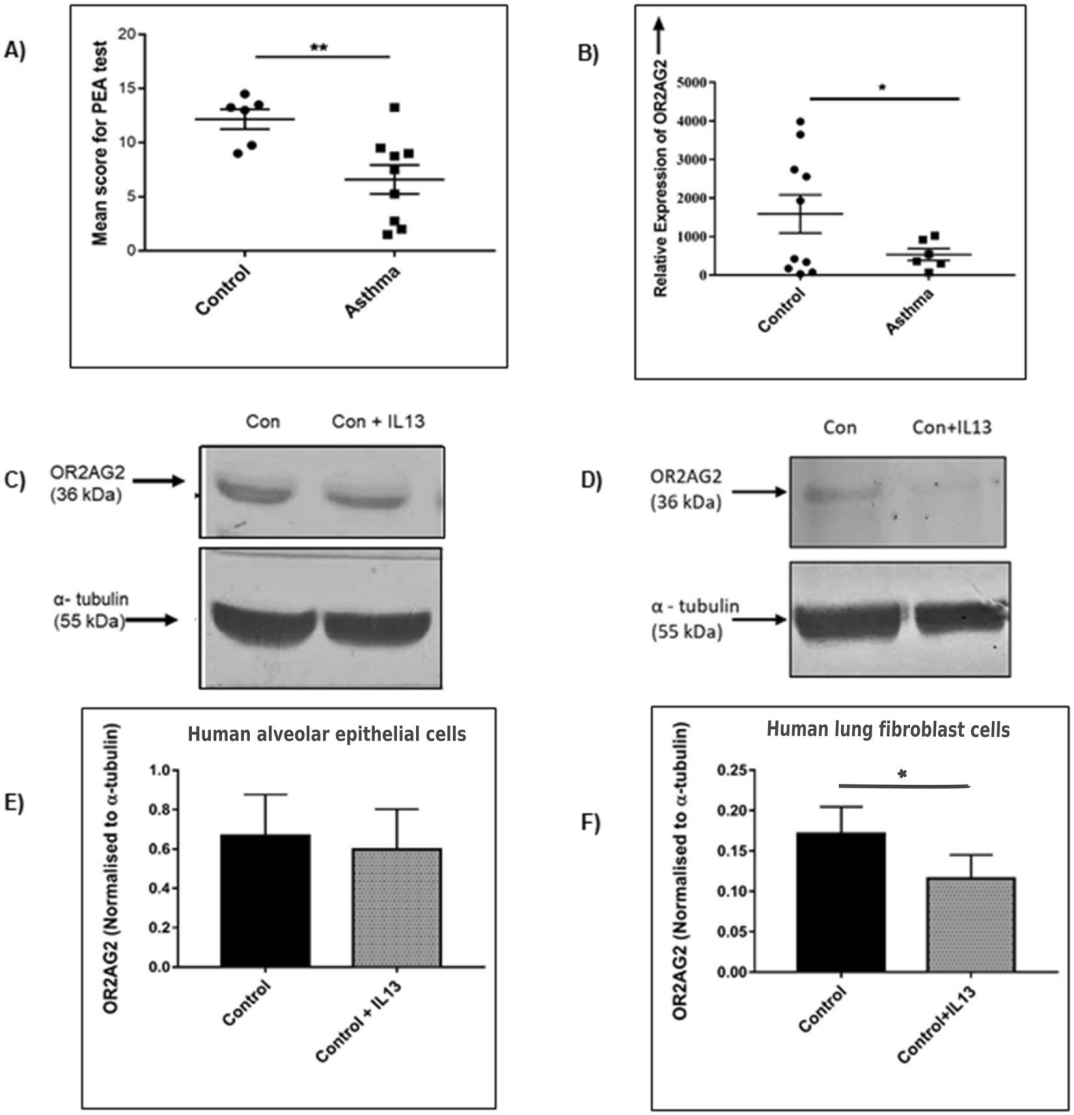
Threshold tests for phenotypic correlation in Family 1, *OR2AG2* levels in human lung samples and *in-vitro* epithelial and fibroblast cells, with or without induction with IL-13. (**A**) Plot showing significant decrease in mean score for odor threshold in subjects with asthma as compared to control subjects from the same family when different concentration of 2-phenylethyl alcohol (PEA) was tested (sweet odor); n = 6 control, n = 9 asthmatic subjects. (**B**) The bar graph represents relative mRNA expression of *OR2AG2* in normal human subjects (control) and asthmatic patients (Asthma), using Real time-PCR., n = 6 asthmatics and n = 10 normal subjects. (**C**) The representative western blot showing a decrease in the protein level of *OR2AG2* in human alveolar epithelial cell line (A549), induced with rIL-13 for 24 hours. (**D**) The representative western blot showing decrease in the protein level of *OR2AG2* in human lung fibroblast cells (HFL1), induced with rIL-13 for 24 hours. (**E**,**F**) Densitometric quantification of the experiment performed in (**C**,**D**) respectively, values are normalized to α-tubulin levels. All results are expressed as the mean ± SEM. *P < 0.05 and **P < 0.01 (t-test).

To determine whether *OR2AG2* genetic variations in the subjects were associated with altered olfactory function or other asthma-related phenotypes, we conducted subjective and objective testing. Affected and unaffected members of the family were subjected to olfactory identification and threshold tests using different concentrations of 2-phenylethyl alcohol (PEA) for sweet odor^10,11^. While the ability to identify and differentiate between the different odors were found to be uncompromised between the cases and controls of Family 1 (data not shown), subjects with asthma were able to detect some odors only at higher concentrations, indicating olfactory dysfunction (Fig. 3A). The affected family members also self-reported a diminished olfactory capacity on direct questioning. Control subjects from the family however showed no such olfactory dysfunction. This finding suggested relative functional hyposmia in patients as compared to the control subjects from the same family.

**Figure 4:**
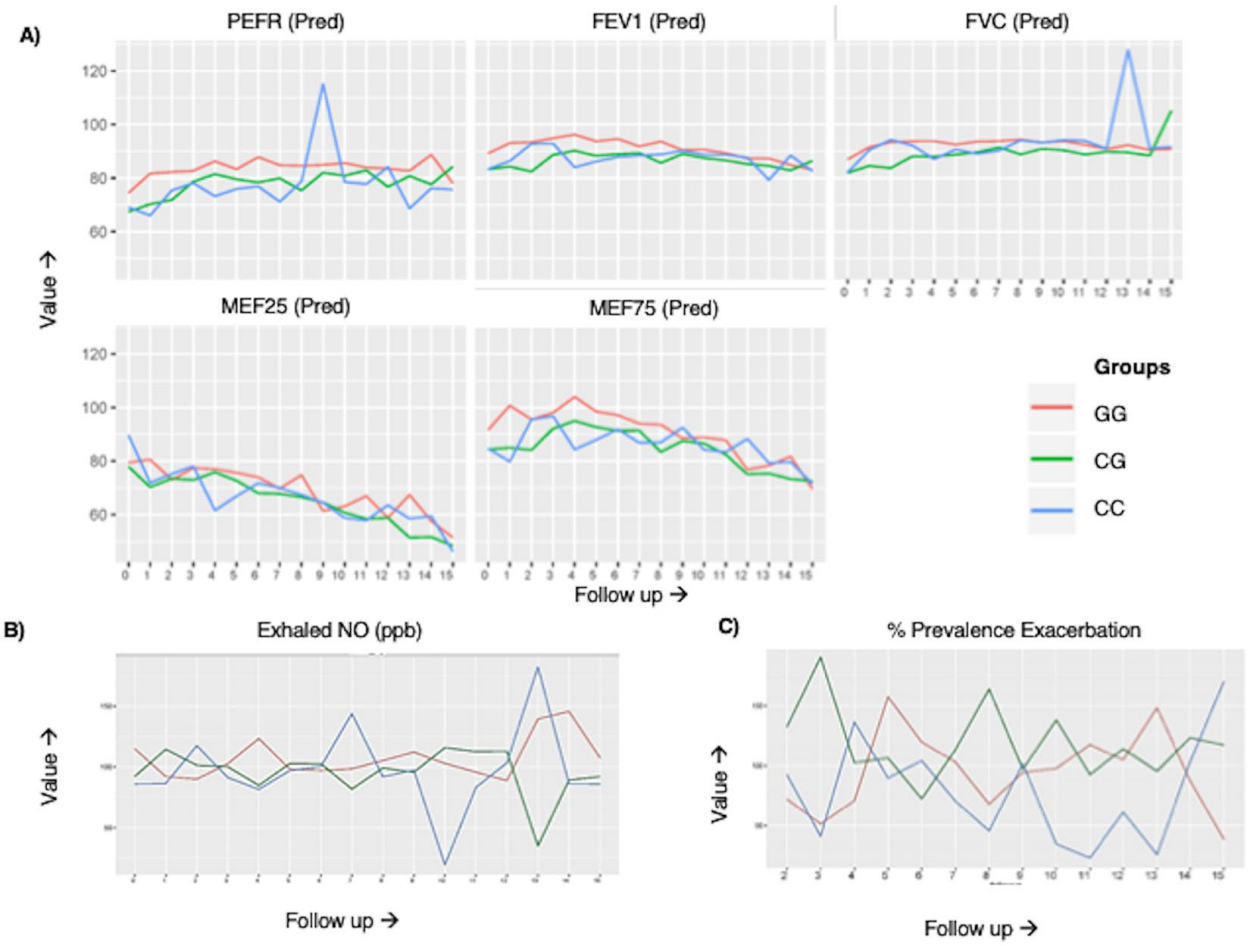
Temporal changes in lung function parameters, exhaled NO and Frequency of exacerbation amongst different genotype asthmatic subjects from the pediatric cohort. (**A**) Line plots showing average percentage of predicted values of different lung function tests for different genotypes (CC, CG and GG) over a period of fifteen follow-ups from baseline amongst asthmatic subjects. (**B**) Line plot showing actual/adjusted exhaled nitric oxide levels in ppb over a period of fifteen follow-ups amongst asthmatic subjects, grouped according to their genotypes. (**C**) Line plot showing actual/adjusted frequency of exacerbation over a period of fifteen follow-ups amongst asthmatic subjects, grouped according to their genotypes.

However, we could not clearly ascertain whether the differences were due to asthma or the genotype. In subjects from the pediatric asthma cohort, for whom genotypes were known, there was a general reduction of olfactory performance comparable to asthmatics from the large family, but there was no significant difference related to genotype. The asthma phenotypes of the three groups were also broadly similar, with no clinically important differences being noted in lung function or other relevant parameters (Table 3, Fig. 4). As an alternate explanation of similarity between asthmatic subjects with or without *OR2AG2* variants, we hypothesized that *OR2AG2* may be suppressed during molecular pathogenesis of asthma. The hypothesis that asthma may be associated with decline in *OR2AG2* was therefore explicitly tested in human samples and *in-vitro* experimental set up with IL-13 induction.

### *OR2AG2* transcripts are significantly lower in lungs of asthmatics and are inhibited by IL-13

To validate our hypothesis, we compared the levels of *OR2AG2* in RNA from lung lysates of asthma patients and normal subjects. OR2AG2 enrichment in lung tissues or resident cells has not been reported in previous studies on olfactory receptors^12–14^. However, in our study, a significant decrease in the level of *OR2AG2* was seen across asthmatics at the RNA level, as measured by real time PCR (Fig. 3B). As expected from the MAF of the variant, the presence of natural variation in the control dataset can be observed from low level of *OR2AG2* in few samples. Asthmatics, on the other hand show consistent decline in the level of *OR2AG2*. We next examined the possibility that *OR2AG2* is downstream of established asthma-related molecular pathways in cell culture studies. Treatment with (recombinant) IL13, a prototype cytokine for asthma^15,16^, led to suppression of *OR2AG2* in human lung cells, as shown in Fig. 3(C–F). Together the data supported the hypothesis that *OR2AG2* may be a convergence point for asthma pathways at lung level.

## Discussion

Asthma, being a well-known heritable complex disease, has been a target for genomic studies for decades. However, focussing on variants of high importance in families, but not frequent enough to explain population level asthma risk, may help in better understanding asthma genetics.

To achieve the same, a large family with four generations affected with asthma was selected and subjected to exome sequencing and whole genome genotyping. Of the validated disease co-segregating genetic variants, one of the variants in gene *OR2AG2* (c.161 G > C, rs10839616) belonging to the family of olfactory receptors, was chosen for further validation for three reasons: 1) it showed significant association with asthma in a small asthma case-control cohort from a different population signifying relevance in general population, 2) patients from the family self-reported inability to sense odors and fumes, and 3) the possibility of the gene’s novel role in asthma biology. This was particularly interesting since the genetic variant was found to be a part of a risk haplotype that segregated with all the affected members of the family in addition to being significantly associated in a separate study on pediatric cohort of asthma (unpublished data from an ongoing project). Our observation that the genetic variant identified from a familial set-up was found to be associated with asthma in a cohort of subjects coming from a different population signifies the relevance of such a study design in the general population. Similar to bitter taste receptors, olfactory receptors are known to be expressed in the airways. They are hypothesized to play role in sensing strong odorant molecules from the environment which in turn can activate ciliary movements for clearance of mucus^8^. This process, when defective, may activate sensory neurons in the airways ultimately triggering exacerbation in asthmatics. Recent reports that suggest that olfactory receptor *OR2AG1* (paralog of *OR2AG2*) are expressed in airway smooth muscle (ASM) cells. While one study shows activation of the receptor leads to histamine induced contraction in ASM cells^17^, other recent reports suggest that its inhibition hinders smooth muscle cell relaxation by triggering transient increase in Ca^2+^ via cAMP dependent signalling cascade^12,18^. We hypothesized that defect in the olfactory receptor gene *OR2AG2* in asthmatics can possibly trigger exacerbation in response to strong odor stimuli, by influencing either the contractility of smooth muscle cells or the mucus clearance ability of the receptor-expressing cells in the lungs and airways.

We observed remarkable phenotypic correlation with our genotypic findings in the family. Most asthmatics would be sensitive to the presence of increased irritants including allergens in the environment and tend to avoid situations where they are increased, since an exposure to higher concentrations of irritants and allergens can lead to severe asthma attacks. Patients from this family self-reported inability to sense odors and fumes, representing a hostile environment, until the content in air is markedly high, after which they experienced severe symptoms and even asthma exacerbations. However, the control subjects of the family remain unaffected. This observation was confirmed by performing odor threshold test using different dilutions of PEA (sweet odor) solution on subjects from family 1. The ability to detect odor was seen to be significantly reduced in asthmatic subjects, demonstrating relative hyposmia as compared to controls from the same family. Although the genotype to phenotype correlation in the cohort was less pronounced, we observed ~80% of the asthmatics from the cohort to carry a copy of the disease allele. While the differences in airflow limitations were modest, these were young asthmatics with mild to moderate forms of asthma. The genotype related differences in the pediatric asthma cohort may emerge over a longer period of time. Additionally, larger sample size of the asthma cohort and more extensive genomic analysis, such as imputation of the non-genotyped variants, can provide further information. It is notable that the index family where the investigation started had drawn clinical attention for the large number of asthmatics as well as more difficult to control asthma. Future investigation follow up of the childhood asthma subjects may also shed more light upon this subject.

Together the data is consistent with a model where either innate genetic defects in *OR2AG2* or acquired cytokine mediated suppression of *OR2AG2* contributes to asthma pathogenesis. Since olfactory function is relevant to the asthma phenotype, with strong odors being known to trigger asthma, the mechanisms may be related to abnormal olfaction or impaired relaxation of airway smooth muscle cells. However, other possibilities cannot be excluded and the relevance of *OR2AG2* in asthma genetics needs to be probed further. To end, our study highlights the value of large family studies in accelerating genetic discovery of complex diseases.

## Methods

### Subjects for the study

A multi-generation family (Family 1) with asthma was identified for this study (Fig. 1). Subjects were diagnosed by a primary physician familiar to the family for generations, based on history of episodic chest symptoms along with pulmonary functions satisfying American Thoracic Society (ATS) criteria^19^. Approximately 40% of the family members were diagnosed with allergic asthma and atopy based on Global Initiative for Asthma (GINA) guidelines. We performed spirometry pre and post bronchodilator treatment for all twenty subjects who provided written consent for the study (Table 1). Subjects with atopy without asthma e.g. allergic rhinitis/dermatitis were excluded from the analysis (III-13, IV-5, IV-29, V21, and V41). In addition to this, 141 asthmatic subjects from a pediatric cohort and 130 controls were used for performing whole genome genotyping. Pediatric subjects were part of an ongoing prospective asthma cohort at All India Institute of Medical Sciences (AIIMS), New Delhi, India, for which multidimensional genotype and phenotype data is being collected. Asthmatic children were recruited based on American Thoracic Society/European Respiratory Society criteria (ATS/ERS)^20^ using patient history, presenting symptoms and spirometric evaluation along with informed consent. Control normal adults were healthy non-smoking volunteers with no history of chest diseases/symptoms and thus chosen for the purpose of representing people with low risk for respiratory disease.

### Informed consent and ethics

Written informed consent was obtained from all participating subjects (in case of children, parent/guardian’s consent was obtained). The nature of the study and its purpose was explained to participants and their parents. The study was designed and performed in accordance with the relevant guidelines approved by the Institutional Human Ethics Committee of CSIR-Institute of Genomics and Integrative Biology, Delhi, India under ethics approval number: IHEC/2015/06 and All India Institute of Medical Sciences (AIIMS) Ethics Committee for the asthma cohort study, with approval number A-15/5.5.2008.

### DNA isolation and exome sequencing

The family pedigree depicted cases of consanguinity but no clear pattern of inheritance. Thus for exome sequencing, affected subjects from distant branches of pedigree were chosen. Detail of sample selection criteria for exome sequencing has been explained in supplementary. DNA was isolated from blood using Qiagen DNA blood mini kit. Eight subjects- II:5, III:7, III:9, III:10, IV:31, IV:34, V:27, V:42 (five cases and three controls) were selected for exome sequencing using the Illumina TruSeq DNA exome kit for the capture of the exonic region as per manufacturer’s protocol. 6 samples were multiplexed and loaded on a single lane of the Hiseq. 2000 flow cell for sequencing. Post sequencing, data was subjected to quality check (QC) and bioinformatics analysis^21^ with minor modifications as described in Supplementary Methods (Supplementary Fig. E1A).

### Variant prioritization

To understand the possible implication of the variants in human physiology and disease, in-silico prediction tools such as SIFT^22^, Polyphen-2^23^, CADD^24^, GERP^25^ and MutationTaster^26^ were exploited to prioritize and select deleterious variants with implications in asthma biology (Supplementary Fig. E1B). Apart from this, variants from genes unreported in asthma (i.e. novel) that co-segregated with all the affected subjects i.e. under disease condition were given priority. The minor allele frequency (MAF) of each variant was obtained using the Exome Variant Server^27^ and 1000Genome variants from the dbSNP database^28^. NHGRI, NCBI, Genecards and SNP4diseases were used to list known asthma genes.

### Confirmation of genetic findings

#### Sanger and SNaPshot sequencing

To remove false positives, genetic variants shortlisted from exome data were confirmed in all twenty members of the family using SNaPshot (for Single nucleotide variations) and Sanger sequencing (for indels) approaches. Primer details are provided in Supplementary Table E1.

#### Whole genome genotyping of family

In DNA from 20 subjects of the family, whole genome genotyping was performed using Illumina Infinium Global Screening Array kit, version 2.0 following manufacturer’s protocol. The genotypes were called using Genome Studio 2.0. Linkage analysis was performed using Haploview^29^ and Haplotype analysis was performed using PHASE tool^30^, version 2.1.1 (shown in Supplementary Fig. E3).

#### Asthma case control cohort

In DNA obtained from an ongoing case-control cohort (as explained in the next sub-topic) of 271 individuals (141 asthmatics and 130 control), whole genome genotyping was performed using IlluminaOmni1-Quad SNP kit following manufacturer’s protocol. Subject details are provided in Table 2. The data was subjected to quality control and analysis using PLINK software^31^. The dataset was used to check the association of the validated variants from exome sequencing to asthma in population.

### Pediatric cohort of asthma

141 subjects from pediatric cohort of asthma at AIIMS subjected to a 3-month follow-up for 5 years. Pediatric subjects were part of an ongoing prospective asthma cohort at All India Institute of Medical Sciences (AIIMS), New Delhi, India, for which multidimensional genotype and phenotype data is being collected (this study was approved by the Ethics Committee of AIIMS). Asthmatic children were recruited based on American Thoracic Society/European Respiratory Society criteria (ATS/ERS)^20^ using patient history, presenting symptoms and spirometric evaluation. Control normal adults were healthy non-smoking volunteers with no history of chest diseases/symptoms and thus chosen for the purpose of representing people with low risk for respiratory disease. Sample details are provided in Table 2. The Lung function values, Exhaled Nitric Oxide (FeNO), Exacerbation frequencies were recorded during these follow-ups. Predicted values for lung function were plotted over the period of 15 follow-ups. Exhaled nitric oxide values (FeNO) and exacerbation frequencies were adjusted for age, gender and height. Actual values for both of them were normalised with the adjusted values. All the plots have been shown in Fig. 4. The cohort characteristics were derived for three genotype groups GG, CG and CC. For comparison of FeNO value and exacerbation frequency between the genotypes, mean value of Actual/adjusted value of FeNO/exacerbation frequencies were computed and plotted for three genotypes from followup-1 to followup-15, respectively, as shown in Table 3 and Fig. 4. The statistical significance for the difference between the genotypes for Lung-function, exhale nitric oxide and exacerbation-frequency was calculated using ANOVA (parametric) and Kruskal-wallis test (non-parametric) after testing for the normality assumptions using Shapiro-Wilk test. The multiple testing correction was performed using Bonferroni adjustment (P- value is ≤α/n), 10 comparisons were made so results were considered to be significant for α = 0.05, when P-value is ≤0.005. All the analysis was carried out using R statistical software.

### Multiple sequence alignment

3D structure of the protein *OR2AG2* was obtained using Chimera tool^32^. Multiple sequence alignment was performed using NCBI blast tool and t-coffee^33^ to investigate the conservation of the protein sequence in the regions flanking the variant of interest.

### Human lung samples

Human lung specimens were obtained with informed consent from patients undergoing thoracic surgery at St. Mary’s Hospital, Mayo Clinic Rochester after approval from institutional review board (from our collaborator Dr. Y.S. Prakash). The patients from whom the samples were derived are Caucasian. Details of human subjects is provided in Supplementary Table E2.

Protocols for cell culture, total cell/tissue lysate, cDNA synthesis, real time PCR and immunoblotting are mentioned in the Online Supplementary.

### Olfactory test

Identification and threshold test using 2-phenylethyl alcohol (PEA, sweet odor) was performed using Sniffin’ Sticks, purchased from Burghart Messtechnik GmbH, Germany. Manufacturer’s protocol was used for the tests^11^. The experimenters were blinded to patient assignment during the tests. Subjects with rhinitis/sinusitis were excluded from the test. Odor score is defined as the tube number at which the subject was able to identify the odorant molecule. The mean score of the odor threshold is an inverse estimate to detect odorant molecules, i.e. lower the mean score in a subject, poorer is their ability to detect odor since activation of the olfactory receptor would require higher concentration of the odorant ligand.

### Statistical analysis

Comparison between two groups were performed using unpaired student’s t-test (parametric) and Wilcoxon’s test (Non-parametric), after testing for normality using Shapiro-Wilk test. Similarly, for comparison between more than two groups, ANOVA or Kruskal wallis was performed based on the normality testing. All data is represented as mean ± SEM, unless stated otherwise.

## Supplementary information

Online Supplement text and figures_Revised

## Data Availability

All results are available in the manuscript or in the supplementary file. Additional information will be available from the corresponding author upon reasonable request.
