# Supplementary material for: Genetic variations in olfactory receptor gene OR2AG2 in a large multigenerational family with asthma
Source: Sci Rep. 2019 Dec 13;9:19029. doi: 10.1038/s41598-019-54718-6 (PMC6911056; doi:10.1038/s41598-019-54718-6)
Supplement: Supplementary file 1 — Online Supplement text and figures_Revised [file 41598_2019_54718_MOESM1_ESM.pdf]

Online Supplementary

Genetic variations in olfactory receptor gene OR2AG2 in a large multigenerational family with asthma.

Samarpana Chakraborty, PhD[1,2], Pushkar Dakle MSc[1], Anirban Sinha, PhD[1], Sangeetha Vishweswaraiah, PhD[3], Aditya Nagori, BE[1,2], Shivalingaswamy Salimath, MD[4], Y. S. Prakash, MD PhD[5], R. Lodha, MD[6], S. K. Kabra, MD[6], Balaram Ghosh, Ph.D.[1,2], Mohammed Faruq, MBBS PhD[1,2], P.A. Mahesh, DNB[4] and Anurag Agrawal, MD PhD[1,2*]

[1]Centre of Excellence for Translational Research in Asthma and Lung Diseases, CSIR-Institute of Genomics and Integrative Biology, New Delhi, India.

[2]Academy of Scientific and Innovative Research (AcSIR), Chennai, India

[3]Genetics and Genomics Lab, Department of Genetics and Genomics, University of Mysore, Manasagangotri, Mysuru, Karnataka, India.

[4]Department of Pulmonary Medicine, JSS Medical College, JSSAHER, Mysore, Karnataka, India

[5]Departments of Anesthesiology, Physiology and Biomedical Engineering, Mayo Clinic, Rochester, Minnesota, USA

[6]Department of Pediatrics, All India Institute of Medical Sciences, New Delhi. India

*Corresponding Author

Dr. Anurag Agrawal, MBBS, Ph.D., FCCP

Director, CSIR- Institute of Genomics and Integrative Biology, New Delhi, India.

Email: a.agrawal@igib.res.in

Methods

Sample selection for exome sequencing:

Blood samples were collected from twenty-five subjects belonging to a multigenerational family in India with physician diagnosed asthma and atopy in more than 40% subjects. Since our study focuses on asthma, subjects with atopy without asthma e.g. allergic rhinitis/dermatitis were excluded (III-13, IV-5, IV-29, V21, and V41). Clinical history along with spirometry pre and post bronchodilator treatment was performed in the remaining twenty subjects, details of which are tabulated in **Table 1**. Power calculation is used for population based genetic studies where statistical significance of the genetic variants increases with sample size. In family based genetic studies, such as ours, the relevance of genetic variants can be observed by their co-segregation with the affected members of the family. Thus, instead of power calculation for selecting subjects for exome sequencing, distant affected relatives and an affected consanguineous couple were chosen to identify clinically relevant variants. This is because distant relatives are expected to share smaller sections of the genome, and therefore, the chances of identifying variants that co-segregate with the disease will be higher. Based on this criteria, eight subjects- II:5, III:7, III:9, III:10, IV:31, IV:34, V:27, V:42 (five cases and three controls) were selected for exome sequencing.

Exome sequencing and bioinformatic analysis of data:

DNA was isolated from blood using Qiagen DNA blood mini kit. Exome sequencing was performed following Illumina's extended exome sequencing protocol on Hiseq 2000. Post sequencing, bcl files obtained from HiSeq are demultiplexed converted to fastq files. The fastq files are subjected to data quality check on a phred scale (Q-score) using FASTQC tool (http://www.bioinformatics.babraham.ac.uk/projects/fastqc/). Phred scale is $-10 \log_{10}$ (error rate). A Q-score of 30 or above signifies good quality reads. To avoid use of erroneous bases due to sequencing errors, 3' end of the reads and adapter sequences are trimmed using Trimmomatic[1]. Post trimming and quality checks, reads are aligned to the human reference genome GRCh37 using BWA and Stampy tools[2,3]. This is followed by picard (http://broadinstitute.github.io/picard/) and samtools[4] to remove PCR duplicates and chromosome wise sorting of the reads. Next step is Indel realignment, base recalibration and variants calling using Genome analysis tool kit (GATK)[5] to generate the vcf file. For annotations SNPeff[6], Seattleseq[7]and Annovar[8] were used. A schematic of the data analysis pipeline is shown in Figure E1.

Segregation of genetic variants

Post exome data analysis, the annotated list of variants was used for comparing the genotype status of variants in affected and unaffected individuals. Since asthma does not follow any particular mode of inheritance and no clear inheritance pattern could be deciphered from the pedigree, a model free approach was used for identifying variants that segregate with cases alone. This means both the conditions were considered: genotype of cases is homozygous and controls is heterozygous and vice versa. The genetic variants that showed consistent pattern of genotype in all the cases barring the controls, were considered to be segregating with disease.

Variant Prioritization

Using online repositories of GWAS data and other catalogues of gene-lists specific to diseases such as NHGRI, NCBI, Genecards and SNP4diseases, a list of known asthma genes was identified. This list was further refined by

removing duplicate entry of genes and the final collated list was used for checking the overlap of known asthma genes with the gene-lists obtained from our exome data. Venn diagram showing the overlap of known asthma genes with genes segregating with cases alone was prepared using Venny tool [9]. 910 variants from 417 genes segregated with the affected subjects. Of these 417 genes, less than 2% were seen to overlap with previously reported asthma genes as shown in Venn diagram (as shown in Figure E2)

To understand the possible implication of the variants in human physiology and disease, in-silico prediction tools were exploited. Tools such as SIFT[10] and Polyphen-2[11] predicts the effect of a variant on protein structure or function, while CADD[12] identifies deleteriousness of a given variant. GERP[13] was used to predict the evolutionary significance of each variant while MutationTaster[14] provides information whether the variant places its effect on UTRs, disrupts splice sites or protein structure/activity or is a harmless synonymous polymorphism. To check the minor allele frequency (MAF) of each variant, information was collected from exome variant server (EVS) from the NHLBI GO exome sequencing project (ESP) and 1000Genome variants from the dbSNP database.

Sanger and SNaPshot sequencing

To remove false positives, genetic variants shortlisted from exome data were confirmed in all twenty members of the family using SNaPshot (for Single nucleotide variations) and Sanger sequencing (for indels) approaches. Sanger and SNaPshot sequencing was performed on ABI 3130 sequencer. Sanger analysis was done using Sequencing Analysis software version 5.1.1. For SNaPshot sequencing, data was visualized on ABI GeneMapper software version 3.5.

Whole genome genotyping

- **Family – Cases and controls**

In DNA from 20 subjects of the family, whole genome genotyping was performed using Illumina Infinium Global Screening Array kit, version 2.0 following manufacturer's protocol. The genotypes were called using Genome Studio 2.0.

Linkage analysis was performed on SNPs with MAF>0.05, spanning ±100kb around variant rs10839616 using Haploview[15] and Haplotype analysis was performed using PHASE tool[16], version 2.1.1 (shown in **Figure E3**). The SNPs that were screened in addition to rs10839616 were rs105147, rs2286163, rs999571, rs1104739, rs36027301, rs113763935, rs192280425, rs117180831, rs61887548, rs2511435, rs10791957, rs117383085, rs61890479, rs1547890, rs4256988, rs1894204, rs75640100, rs4930561, rs74625804, rs75596059, rs117298389, rs10896300, rs80146147 and rs202232579.

- **Asthma case control cohort**

In DNA obtained from an ongoing case-control cohort of 271 individuals (141 asthmatics and 130 control), whole genome genotyping was performed using Illumina Omni1-Quad SNP kit following manufacturer's protocol. The genotypes were called using Genome Studio 2.0 and the data was subjected to quality control and analysis using PLINK software[17]. The dataset was used to check the association of the validated variants from exome sequencing to asthma in population. Of the final 26 validated variants identified by exome sequencing, one variant i.e. rs10839616 was found to be present in the asthma case-control cohort.

Additionally, to cross validate our findings, an online database portal: GWAS Central was checked to identify overlap, if any, with previously published large GWAS studies/metanalysis of GWAS studies. Few of our final variants (n=26) were observed to be reported in previous asthma and lung studies. The findings have been tabulated in **Table E3**.

Statistical analysis

Comparison between two groups were performed using unpaired student's t-test (parametric) and Wilcoxon's test (Non-parametric), after testing for normality using Shapiro-Wilk test. Similarly, for comparison between more than two groups, ANOVA or Kruskal wallis was performed based on the normality testing. All data is represented as mean ± SEM, unless stated otherwise.

Cell lines

Cells were purchased from ATCC. Adenocarcinoma human lung basal epithelial cells, A549 were cultured in DMEM-HG (Sigma) and Human lung fibroblast cells, HFL1 were cultured in DMEM-LG (D5523): Hams F12 (56659C) in 1:1 ratio. Cells were maintained at 37°C in a humidified atmosphere with 5% CO_2. Recombinant IL13 (rIL-13) was purchased from R&D systems and used at 20ng/ml concentration. Serum starved A549/ HFL1 cells were induced with or without human rIL-13 for 24 hours after which cells were harvested.

Total cell lysate (TCL) preparation

24 hours post induction with or without rIL13, cells were trypsinized from 6-well plate or T-25 flasks, pelleted and washed with 1X PBS. Post centrifugation, the pellet was resuspended in RIPA buffer (containing 1 mM DTT and cocktail of protease inhibitors), incubated and centrifuged again at 15,000 rpm for 45 min at 4° C. Finally the supernatant was collected as total cell extract and stored at -80°C till further use.

Human Lung Samples

Human lung specimens were obtained from patients undergoing thoracic surgery at St. Mary's Hospital, Mayo Clinic Rochester, MN (from our collaborator Dr. Y.S. Prakash) Sample details are provided in **Table E2.** Briefly, under Mayo's Institutional Review Board-approved protocols, third- to sixth-level bronchi from human lung specimens were obtained. Based on patient histories, age matched asthma and normal samples were used. A written informed consent was obtained from the participants and the Review Board of IGIB, Delhi, India, approved the studies.

cDNA synthesis and Real time PCR

For preparation of lung lysate, 30 mg lung tissue from healthy and asthmatic subjects (obtained from Mayo Clinic) was snap-frozen in liquid nitrogen, followed by crushing the frozen tissue using Qiagen RNA lysis buffer (samples were kept on ice to avoid degradation). After this step, Qiagen RNeasy kit was followed as per the manufacturer's protocol. 1-2 µg of RNA were used to prepare the cDNA by using High-Capacity cDNA Reverse Transcription Kit (Applied Biosystems) as manufacturer's protocol. RT-PCR for OR2AG2 was performed by using kappa SYBR green using Roche instrument (LightCycler 480, USA). RNA from human lung tissue was isolated by RNeasy mini kit (Qiagen). Initially 50mg of lung tissue was subjected to snap-freeze by adding liquid nitrogen and then minced by adding 350 µl of RLT buffer. Post tissue lysis, the rest of the protocol was followed as per manufacturer's instruction. 1-2µg of RNA were used to prepare the cDNA by using High-Capacity cDNA Reverse Transcription

Kit (Applied Biosystems) as manufacturer's protocol. RT-PCR for OR2AG2 was performed using kappa SYBR green using Roche instrument (LightCycler 480, USA).

Immunoblotting

Protocol for immunoblotting has been followed as described previously,[18]. Primary antibody for OR2AG2 (Cell Signalling). α-tubulin/ β-actin (Sigma) was used as loading controls. Full length blots are provided here **Figure E5**.

__Online Repository Figure Legends__

Figure E1 Schematic of the data analysis workflow

A) **Exome sequencing**: bcl files obtained from HiSeq are demultiplexed converted to fastq files. The fastq files are subjected to data quality check on a phred scale (Q-score) using FASTQC tool. Phred scale is -10 $\log_{10}$ (error rate). A Q-score of 30 or above signifies good quality reads. To avoid use of erroneous due to sequencing errors, 3' end of the reads and adapter sequences are trimmed using Trimmomatic. Post trimming and quality checks, reads are aligned to the human reference genome GRCh37 using BWA and Stampy tools and then picard and samtools are used to remove PCR duplicates and chromosome wise sorting of the reads. This is followed by Indel realignment and variants calling using GATK to generate the vcf file. For annotations SNPeff, Seattleseq and Annovar have been used. B) **Variant Prioritisation –** To narrow down the list of annotated variants to identify genetic variants that belong to novel genes in asthma and also predicted to be deleterious, filters were used at each step as described in the given flowchart.

Figure E2 Overlap between known asthma genes and gene-list obtained from families.

A) Venn diagram showing that only a fraction of genes known to asthma are represented in the given family. Of the total 1513 known asthma genes, the number of overlapping genes in Family 1 was found to be 35. B) Shows the list of genes that overlaps between the known asthma genes and gene list from family1.

Figure E3 Haplotype analysis in Family 1.

A) Six variants from chromosome 11p15.4, including rs10839616, were found to belong to a haplotype within Family 1 and also in linkage disequilibrium. A branch from the pedigree has been shown for representation. The alleles for risk haplotype are enclosed in box showing the enrichment of the risk allele for rs10839616 (marked in red) in the affected members of Family 1. B) The LD plot shown in online repository Figure E4B highlights in blue the SNPs that are in linkage disequilibrium with our variant of interest i.e. rs10839616 (demarcated by red arrow) with neighboring SNPs - rs11041009, rs2595498 and rs593313 showing strong LD (D′= 1). Due to limited number of samples, the LD plot shows gaps and could not be resolved further.

Figure E4 OR2AG2 variant information

A) 3D structure of OR2AG2 protein showing the position of variant p.Arg54Pro marked in red. B) The image depicts conservation of the wild type amino acid in region flanking the variant of interest across different species, when subjected to multiple sequence alignment suggesting evolutionary significance. C) In silico prediction of the variant rs10839616 indicate potential loss of function of the receptor, outlined in red.

Figure E5 Full length western blot of OR2AG2 protein levels, shown in Figure 3 (C and D)

The lanes shown in Figure 3 (C and D) are marked by red boxes.

<u>Tables</u>:

Table E1: List of all primers used

S.No.	Primer Name	Oligo Sequence (5' to 3')
1.	F1_FP_BBOX1:	GAGGCCAGAGTCCACTTGAA
2.	F1_RP_BBOX1:	GCACGGACAGTTGTCTCTCA
3.	F1_SP_BBOX1:	TGATGCAGATCCTCTGGTAT
4.	F1_FP_CNN2_rs200177867	AAGGTCCCCTCTTCTCTCCA
5.	F1_RP_CNN2_rs200177867	GGGCAGTACTTGGGGTCATA
6.	F1_SP_CNN2_rs200177867	CTCCTCCATGTCCCTGCAGA
7.	F1_FP_S_CNN2_rs371146424	AAGGTCCCCTCTTCTCTCCA
8.	F1_RP_S_CNN2_rs371146424	GGGCAGTACTTGGGGTCATA
9.	F1_FP_GALNT8	GTGTTGCCGTGTGTTTTGTC
10.	F1_RP_GALNT8	TCACGTATCACGGAGTCCAG
11.	F1_SP_GALNT8	TCTGCTTGGATCAGGGACCC
12.	F1_FP_HLA-A	GTCTGGGTTCTGTGCTCTCTTC
13.	F1_RP_HLA-A	GTGGCCTCATGGTCAGAGAT
14.	F1_SP_HLA-A	TCTGACTCTTCCCGTCAGAC
15.	F1_FP_MUC16_rs11085777	GCAGGGCTTGTGTCTCTAGG
16.	F1_RP_MUC16_rs11085777	CTGCCATTCACCCTCAATTT
17.	F1_SP_MUC16_rs11085777	CCTCCTCATACTGCAGGTTA
18.	F1_FP_MUC16_9008248	CAACCACATCACAGCCACTC
19.	F1_RP_MUC16_9008248	CCACCACCTTAACCCTCAAA
20.	F1_SP_MUC16_9008248	ATTGGTCATCTGGCTCAGCT
21.	F1_FP_MUC2	GGGATTCTGGCTCTTTCTGA
22.	F1_RP_MUC2	CTCGCAGTCATTGGTGATCT
23.	F1_SP_MUC2	GCCACTGCAGGCTGCACGGA
24.	F1_FP_NELL1	TGCAGAAGGACCTAAATGTGG
25.	F1_RP_NELL1	GGATTGCATAGGACCACAGC
26.	F1_SP_NELL1	AAGCTACTTGTGAGTGCAAG
27.	F1_FP_PABPC1_rs113574896	AGCGCCACAGGTTATTATGC
28.	F1_RP_PABPC1_rs113574896	ATCACTGGCATGTTGTTGGA
29.	F1_SP_PABPC1_rs113574896	AGACTCGAGCATATGAAGAA
30.	F1_FP_PABPC1_rs78407297	CCCCATAAAAAGCCACGTAA
31.	F1_RP_PABPC1_rs78407297	ATTCAAGCCATGCACCCTAC

S.No.	Primer Name	Oligo Sequence (5' to 3')
32.	F1_SP_PABPC1_rs78407297	CAACATGCCAGTGATTTTAC
33.	F1_FP_PRSS1	GGTGACCCTCACCTCACAGT
34.	F1_RP_PRSS1	AGGCAGGGCATGATACTCAC
35.	F1_SP_PRSS1	TTACCTTTGTGGCAGCTGCT
36.	F1_FP_SIRT3	AGATCACCCCAACAGCAAAC
37.	F1_RP_SIRT3	TGGCTTGAATTTCAGTGTCG
38.	F1_SP_SIRT3	GAATGTCCTCCCCTGGGAAG
39.	F1_FP_S_SLC25A59_A	CCACATCCCTGTGTTTTGTG
40.	F1_RP_S_SLC25A59_A	CCCTGCACAGACACGTTAAA
41.	F1_FP_S_SLC25A59_B	TTTTTCATCGGCCTTCAGTC
42.	F1_RP_S_SLC25A59_B	TGTTCACAGGGGGAAAAATC
43.	F1_FP_S_TBC1D10C	AAGACTTTCCATGGGCTCCT
44.	F1_RP_S_TBC1D10C	CCCCGAGTCCCAAGAGTAA
45.	F1_FP_WRN	CCTGAAAACATTGACACGTACC
46.	F1_RP_WRN	TGCCTACTTCTTCCTTGCTTCT
47.	F1_SP_WRN	ACAGCGGACTTCAACCTTCA
48.	F1_FP_OR13C5	TGGCATCCAAGTCATCTGAA
49.	F1_RP_OR13C5	GGCAATGAGTTCATCCTGCT
50.	F1_SP_OR13C5	GAGACTTGGGCTTCATGTAC
51.	F1_FP_OR1L6	CTGCTCTGACACATCCTCCA
52.	F1_RP_OR1L6	GTACATGGACAGGGGCCTAA
53.	F1_SP_OR1L6	TGTGACCCCCTTCCTGTGTA
54.	F1_FP_OR2AG2	TGCCAGGAACATCTGAAGTG
55.	F1_RP_OR2AG2	TGGGTCTCCTGAACTGCTCT
56.	F1_SP_OR2AG2	GGTACATGGGCATGTGGAGC
57.	F1_FP_OR51I1_A	TTCACCACAAAAGGGAAAGG
58.	F1_RP_OR51I1_A	GCCTGGTCCAGATGTTCTTC
59.	F1_SP_OR51I1_A	GATAACAAATAGCCACAAAG
60.	F1_FP_OR51I1_B	GCCTGTTTGTATCCCAGGAA
61.	F1_RP_OR51I1_B	TCTTACTGGGAAGGCAGGAA
62.	F1_SP_OR51I1_B	CCCAGGAATGCCTGTCAGCT
63.	F1_FP_OR51Q1	TCCATTATGCCTCCATCCTC
64.	F1_RP_OR51Q1	GAGCAAGGCAAGAGCAAATC
65.	F1_SP_OR51Q1	CCACCTTCTCTCTCGCTCCT
66.	OR2AG2_RT_FP	AGGAATAAGGAGGTCATGCG
67.	OR2AG2_RT_RP	GTGAGAGGCAAGCCATGAT

[FP: Forward Primer; RP: Reverse Primer; SP: Snapshot Primer; RT_FP/RP: Primer for Real time PCR]

Table E2: Age and gender details of human subjects for lung samples.

Lung Samples	Males	Females	Age (Mean)
Control	4	6	61.7
Asthmatic	1	5	58.6

Table E3: Overlap of variants from the present study with previously published asthma GWAS reports

**Variant**	**Gene**	**Study**	**Phenotypes Tested**	**p-value**	**GWAS Central ID**	**PMID**
rs1346044	WRN	GWAS of height-adjusted highest forced expiratory volume in a British population	Height-adjusted highest forced expiratory volume	0.02	HGVST310	17255346
rs1346044	WRN	GWAS of asthma	Asthma<br>Late onset asthma<br>Childhood Onset Asthma	0.01	HGVST631	20860503
rs1851724	OR13C5	GWAS of asthma	Asthma<br>Late onset asthma<br>Childhood Onset Asthma	0.6	HGVST631	20860503
rs1851724	OR13C5	GWAS of height-adjusted highest forced expiratory volume in a British population	Height-adjusted highest forced expiratory volume	0.6	HGVST310	17255346
rs10839616	OR2AG2	GWAS of pulmonary function	Pulmonary function (FEV1/FVC)	0.01	HGVST946	21946350
rs16930982	OR51l1	GWAS of pulmonary function	Pulmonary function (FEV1/FVC)	0.65	HGVST946	21946350
rs16930998	OR51l1	GWAS of pulmonary function	Pulmonary function (FEV1/FVC)	0.45	HGVST946	21946350
rs1468552	GALNT8	GWAS of asthma	Asthma<br>Late onset asthma<br>Childhood Onset Asthma	0.003	HGVST631	20860503
rs1468552	GALNT8	GWAS of height-adjusted highest forced expiratory volume in a British population	Height-adjusted highest forced expiratory volume	0.02	HGVST310	17255346

References:

1 Bolger, A. M., Lohse, M. & Usadel, B. Trimmomatic: a flexible trimmer for Illumina sequence data. *Bioinformatics* **30**, 2114-2120, doi:10.1093/bioinformatics/btu170 (2014).

2 Lunter, G. & Goodson, M. Stampy: a statistical algorithm for sensitive and fast mapping of Illumina sequence reads. *Genome Res* **21**, 936-939, doi:10.1101/gr.111120.110 (2011).

3 Li, H. & Durbin, R. Fast and accurate short read alignment with Burrows-Wheeler transform. *Bioinformatics* **25**, 1754-1760, doi:10.1093/bioinformatics/btp324 (2009).

4 Li, H. *et al.* The Sequence Alignment/Map format and SAMtools. *Bioinformatics* **25**, 2078-2079, doi:10.1093/bioinformatics/btp352 (2009).

5 DePristo, M. A. *et al.* A framework for variation discovery and genotyping using next-generation DNA sequencing data. *Nat Genet* **43**, 491-498, doi:10.1038/ng.806 (2011).

6 Cingolani, P. *et al.* A program for annotating and predicting the effects of single nucleotide polymorphisms, SnpEff: SNPs in the genome of Drosophila melanogaster strain w1118; iso-2; iso-3. *Fly (Austin)* **6**, 80-92, doi:10.4161/fly.19695 (2012).

7 Ng, S. B. *et al.* Targeted capture and massively parallel sequencing of 12 human exomes. *Nature* **461**, 272-276, doi:10.1038/nature08250 (2009).

8 Wang, K., Li, M. & Hakonarson, H. ANNOVAR: functional annotation of genetic variants from high-throughput sequencing data. *Nucleic Acids Res* **38**, e164, doi:10.1093/nar/gkq603 (2010).

9 Oliveros, J. C. Venny. An interactive tool for comparing lists with Venn's diagrams. (2007-2015). <http://bioinfogp.cnb.csic.es/tools/venny/index.html%3E.

10 Kumar, P., Henikoff, S. & Ng, P. C. Predicting the effects of coding non-synonymous variants on protein function using the SIFT algorithm. *Nature protocols* **4**, 1073-1081, doi:10.1038/nprot.2009.86 (2009).

11 Adzhubei, I. A. *et al.* A method and server for predicting damaging missense mutations. *Nature methods* **7**, 248-249, doi:10.1038/nmeth0410-248 (2010).

12 Kircher, M. *et al.* A general framework for estimating the relative pathogenicity of human genetic variants. *Nature genetics* **46**, 310-315, doi:10.1038/ng.2892 (2014).

13 Davydov, E. V. *et al.* Identifying a high fraction of the human genome to be under selective constraint using GERP++. *PLoS computational biology* **6**, e1001025, doi:10.1371/journal.pcbi.1001025 (2010).

14 Schwarz, J. M., Rodelsperger, C., Schuelke, M. & Seelow, D. MutationTaster evaluates disease-causing potential of sequence alterations. *Nature methods* **7**, 575-576, doi:10.1038/nmeth0810-575 (2010).

15 Barrett, J. C., Fry, B., Maller, J. & Daly, M. J. Haploview: analysis and visualization of LD and haplotype maps. *Bioinformatics* **21**, 263-265, doi:10.1093/bioinformatics/bth457 (2005).

16 Stephens, M. & Scheet, P. Accounting for decay of linkage disequilibrium in haplotype inference and missing-data imputation. *Am J Hum Genet* **76**, 449-462, doi:10.1086/428594 (2005).

17 Purcell, S. *et al.* PLINK: a tool set for whole-genome association and population-based linkage analyses. *American journal of human genetics* **81**, 559-575, doi:10.1086/519795 (2007).

18 Khanna, K. *et al.* Secretory Inositol Polyphosphate 4-Phosphatase Protects against Airway Inflammation and Remodeling. *American journal of respiratory cell and molecular biology* **60**, 399-412, doi:10.1165/rcmb.2017-0353OC (2018).

Online Supplementary figures

Figure E1

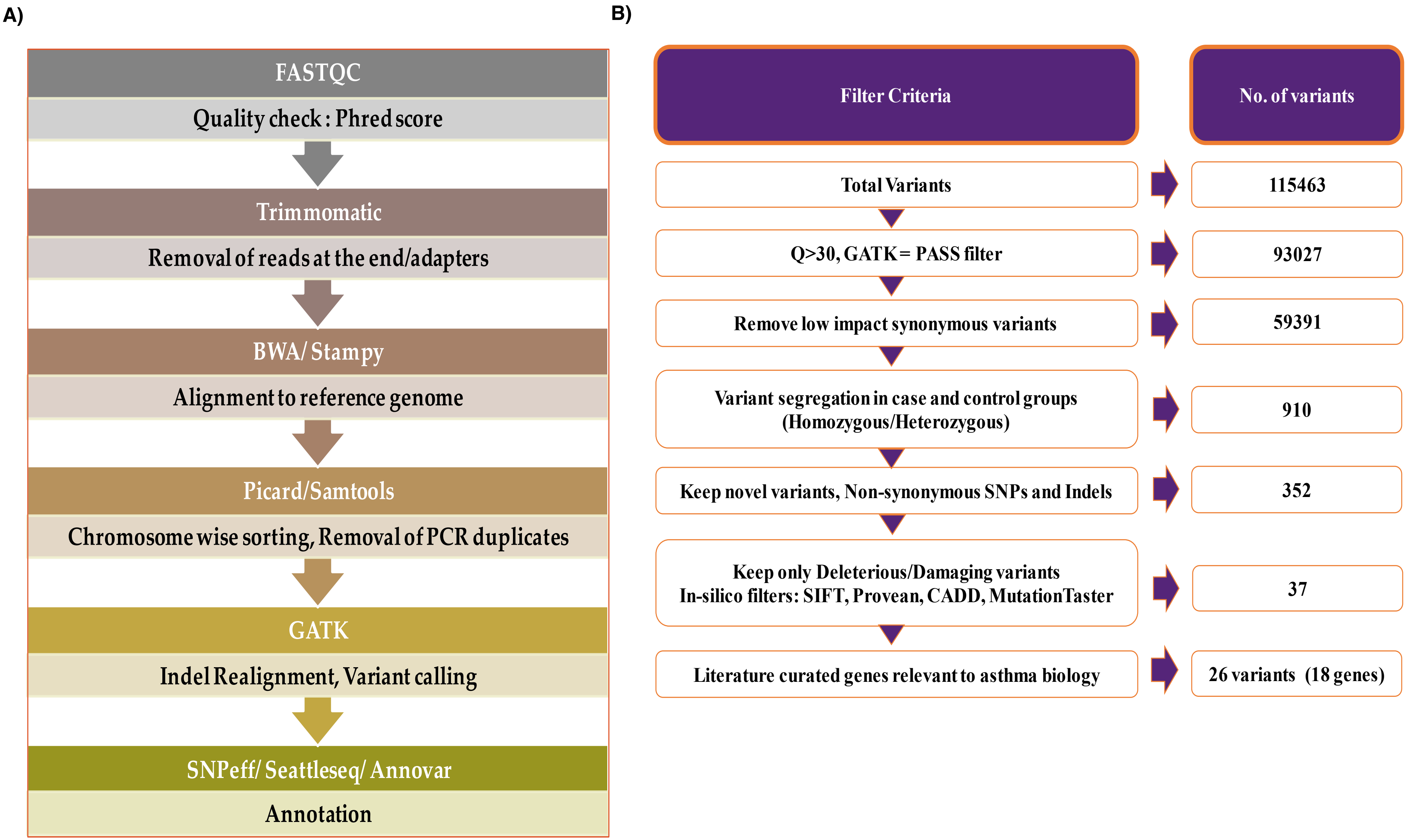
A)
FASTQC
Quality check : Phred score
Trimmomatic
Removal of reads at the end/adapters
BWA/ Stampy
Alignment to reference genome
Picard/Samtools
Chromosome wise sorting, Removal of PCR duplicates
GATK
Indel Realignment, Variant calling
SNPeff/ Seattleseq/ Annovar
Annotation
B)
Filter Criteria
No. of variants
Total Variants
115463
Q>30, GATK= PASS filter
93027
Remove low impact synonymous variants
59391
Variant segregation in case and control groups (Homozygous/Heterozygous)
910
Keep novel variants, Non-synonymous SNPs and Indels
352
Keep only Deleterious/Damaging variants
In-silico filters: SIFT, Provean, CADD, MutationTaster
37
Literature curated genes relevant to asthma biology
26 variants (18 genes)

Figure E2

A)

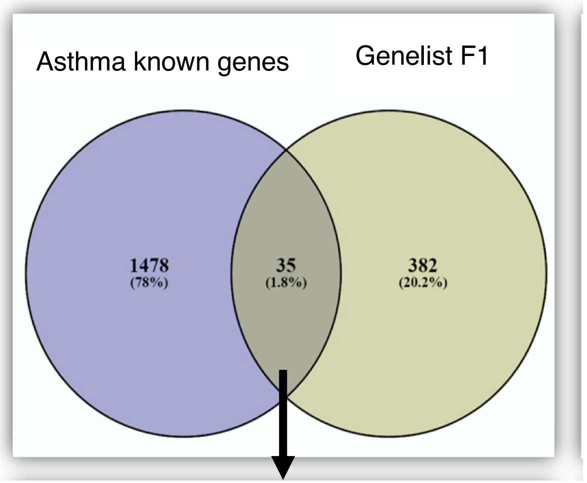

B)

Family 1	
ADAM33	PDE11A
AICDA	PDE4D
ALOX12	PTGER3
C10orf11	PTPRD
CD69	RAB4B
CLCA1	SETDB2
EGFR	SMAD3
EPHX2	SOD2
FAM13A	STAT3
HAVCR1	TNS1
HDAC4	
HHIP	
HLA-A	
HLA-DQA2	
HLA-DQB1	
HLA-DRB1	
IL19	
IL2	
IL4R	
LRP1	
MMP15	
MUC2	
NOS2	
NOX1	

Figure E3

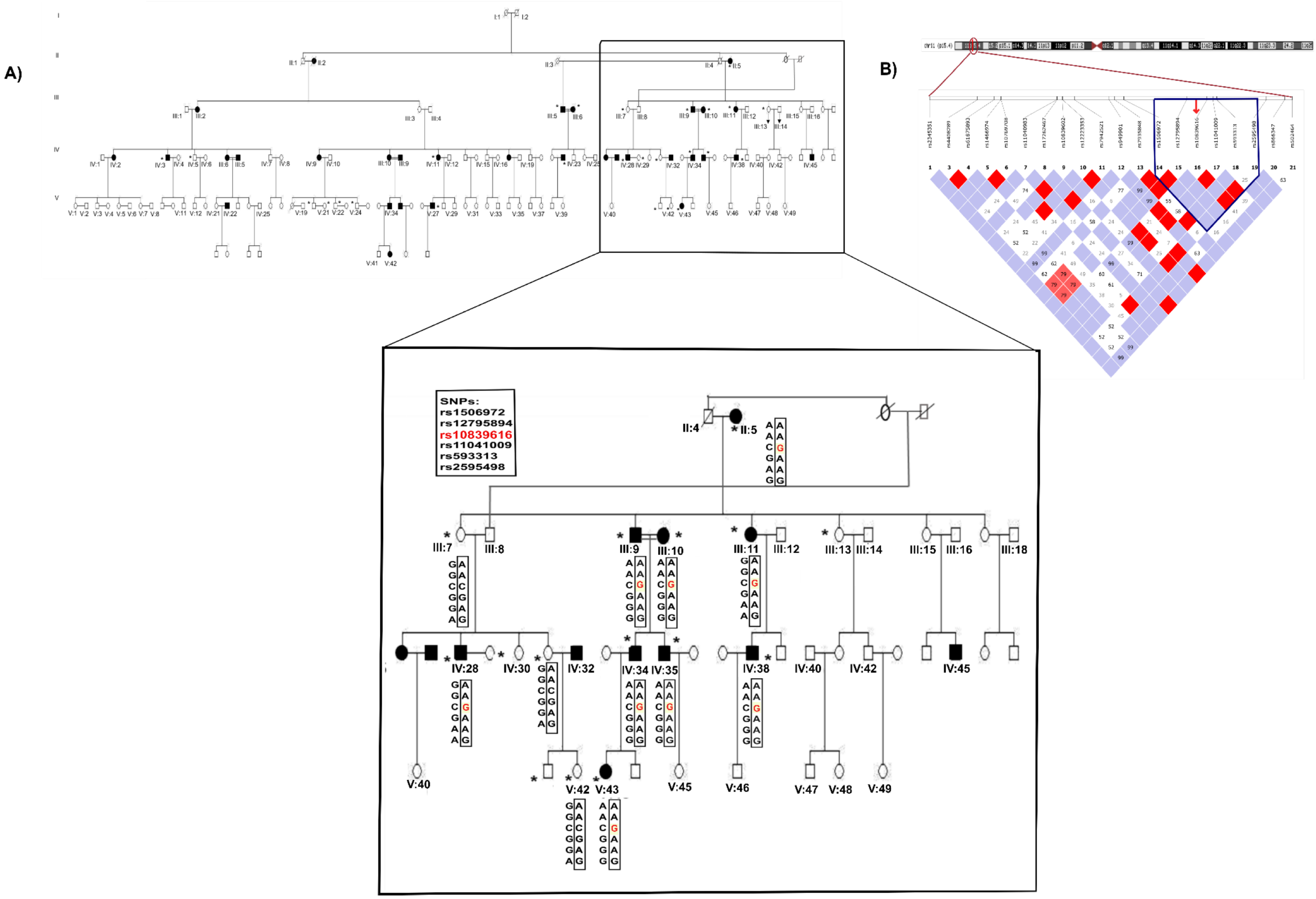
A)
B)
SNPs:
rs1506972
rs12795894
rs10839616
rs11041009
rs593313
rs2595498
II:4
II:5
III:7
III:8
III:9
III:10
III:11
III:12
III:13
III:14
III:15
III:16
III:18
IV:28
IV:30
IV:32
IV:34
IV:35
IV:38
IV:40
IV:42
IV:45
V:40
V:42
V:43
V:45
V:46
V:47
V:48
V:49

Figure E4

A)

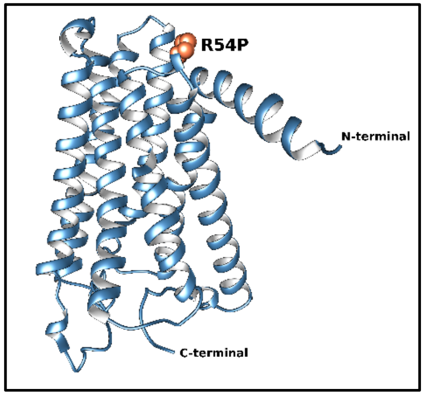

B)

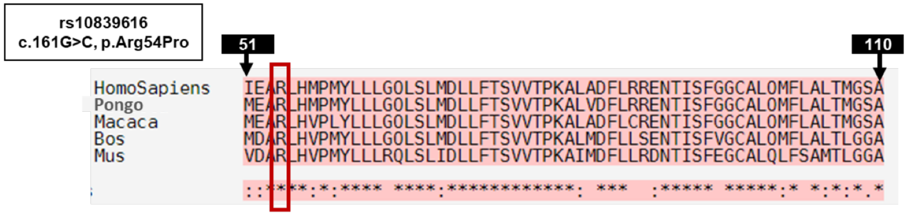

C)

Summary

- amino acid sequence changed
- homozygous in TGP or ExAC
- protein features (might be) affected
- splice site changes

analysed issue

name of alteration	no title
alteration (phys. location)	chr11:6790028C>G show variant in all transcripts IG
HGNC symbol	OR2AG2
Ensembl transcript ID	ENST00000338569
Genbank transcript ID	NM_001004490
UniProt peptide	A6NM03
alteration type	single base exchange
alteration region	CDS
DNA changes	c.161G>C<br>cDNA.259G>C<br>g.259G>C
AA changes	R54P Score: 103 explain score(s)

Protein features

start (aa)	end (aa)	feature	details	
52	56	TOPO_DOM	Cytoplasmic (Potential).	lost
57	77	TRANSMEM	Helical; Name=2; (Potential).	might get lost (downstream of altered splice site)
78	97	TOPO_DOM	Extracellular (Potential).	might get lost (downstream of altered splice site)
97	97	DISULFID	By similarity.	might get lost (downstream of altered splice site)
98	118	TRANSMEM	Helical; Name=3; (Potential).	might get lost (downstream of altered splice site)
119	139	TOPO_DOM	Cytoplasmic (Potential).	might get lost (downstream of altered splice site)
140	160	TRANSMEM	Helical; Name=4; (Potential).	might get lost (downstream of altered splice site)
161	205	TOPO_DOM	Extracellular (Potential).	might get lost (downstream of altered splice site)
179	179	DISULFID	By similarity.	might get lost (downstream of altered splice site)
206	226	TRANSMEM	Helical; Name=5; (Potential).	might get lost (downstream of altered splice site)
227	244	TOPO_DOM	Cytoplasmic (Potential).	might get lost (downstream of altered splice site)
245	265	TRANSMEM	Helical; Name=6; (Potential).	might get lost (downstream of altered splice site)
266	271	TOPO_DOM	Extracellular (Potential).	might get lost (downstream of altered splice site)
272	292	TRANSMEM	Helical; Name=7; (Potential).	might get lost (downstream of altered splice site)
293	316	TOPO_DOM	Cytoplasmic (Potential).	might get lost (downstream of altered splice site)

Figure E5

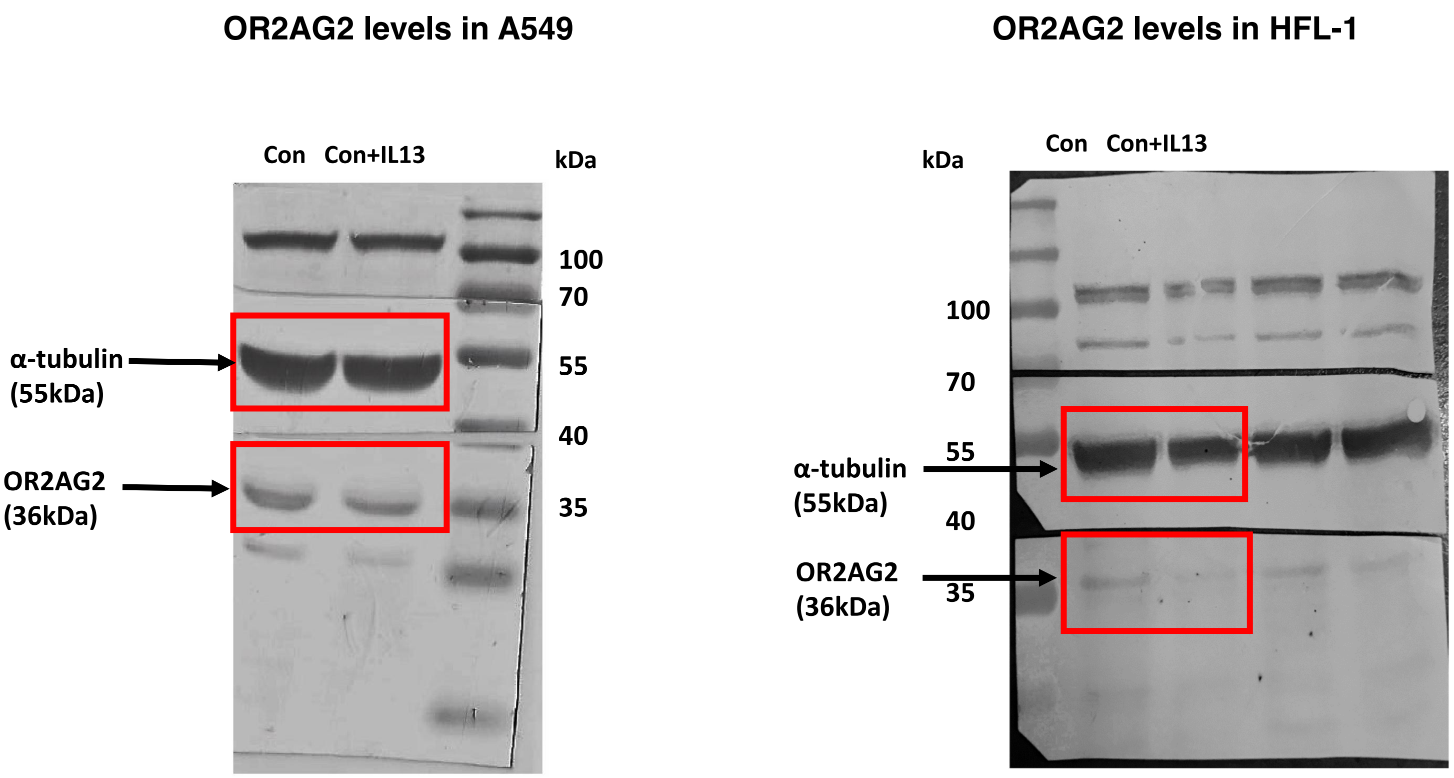
OR2AG2 levels in A549
Con
Con+IL13
kDa
100
70
55
40
35
α-tubulin
(55kDa)
OR2AG2
(36kDa)
OR2AG2 levels in HFL-1
Con
Con+IL13
kDa
100
70
55
40
35
α-tubulin
(55kDa)
OR2AG2
(36kDa)
